# Supplementary material for: Structural basis of CXC chemokine receptor 1 ligand binding and activation
Source: Nat Commun. 2023 Jul 11;14:4107. doi: 10.1038/s41467-023-39799-2 (PMC10336096; doi:10.1038/s41467-023-39799-2)
Supplement: Supplementary file 1 — Supplementary Information [file 41467_2023_39799_MOESM1_ESM.pdf]

Structural basis of CXC chemokine receptor 1 ligand binding and activation

Naito Ishimoto[1†], Jae-Hyun Park[1†], Kouki Kawakami[2], Michiko Tajiri[3], Kenji Mizutani[1], Satoko Akashi[3], Jeremy R.H. Tame[1], Asuka Inoue[2], Sam-Yong Park[1*]

[1]Drug Design Laboratory, Graduate School of Medical Life Science, Yokohama City University, Tsurumi, Yokohama 230-0045, Japan.

[2]Graduate School of Pharmaceutical Sciences, Tohoku University, Sendai 980-8578, Japan.

[3]Structural Epigenetics Laboratory, Graduate School of Medical Life Science, Yokohama City University, Tsurumi, Yokohama 230-0045, Japan.

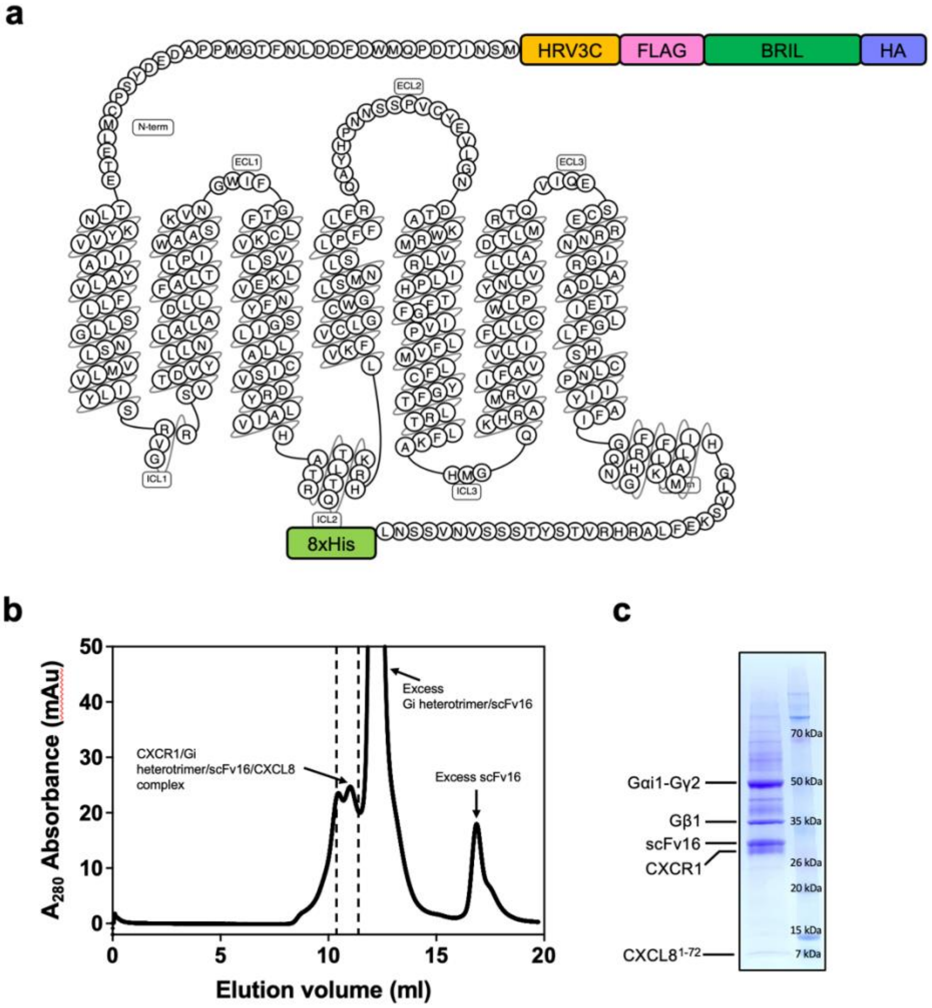

Supplementary Figure 1. **CXCR1 construct and purification of the CXCR1/Gi complex.**

(a) Snake plot of the CXCR1 construct used. (b) Representative size-exclusion chromatography profile of CXCR1/Gi/CXCL8$^{1-72}$ complex. (c) Representative SDS–PAGE gel of CXCR1/Gi/CXCL8$^{1-72}$ complex used for cryo-EM grid preparation, stained with Coomassie-blue. Purification was performed two times for CXCR1/Gi complex with similar result.

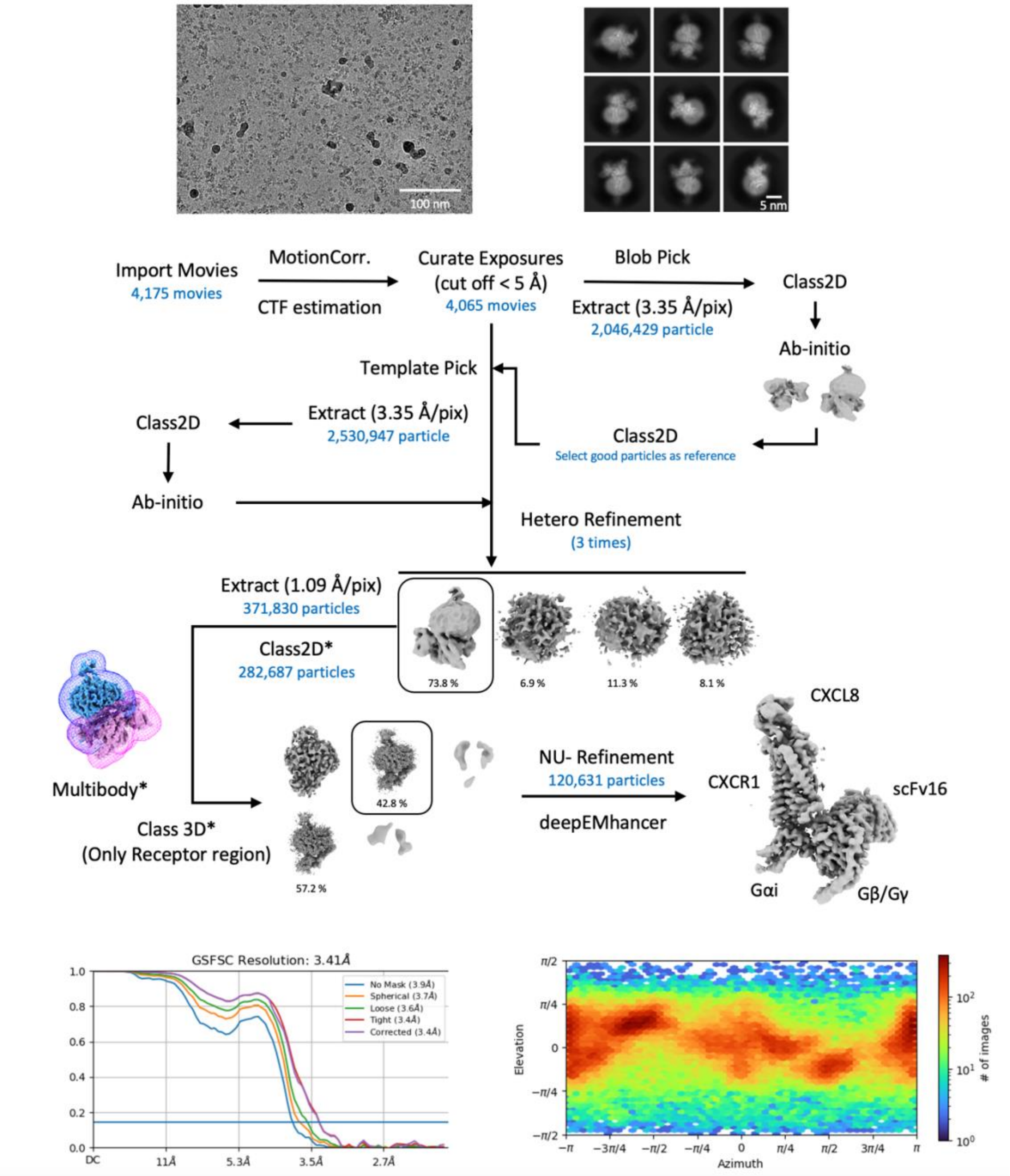

Supplementary Figure 2. **Cryo-EM data collection and processing.**
Cryo-EM data processing workflow for CXCR1/Gi/CXCL8$^{1\text{-}72}$ complex. Representative micrographs and 2D classes of the complexes are shown. Gold-standard FSC curves indicate overall nominal resolution of 3.4 Å using the FSC = 0.143 criterion. Cryo-EM data processing was performed one time for CXCR1/Gi/CXCL8$^{1\text{-}72}$ complex.

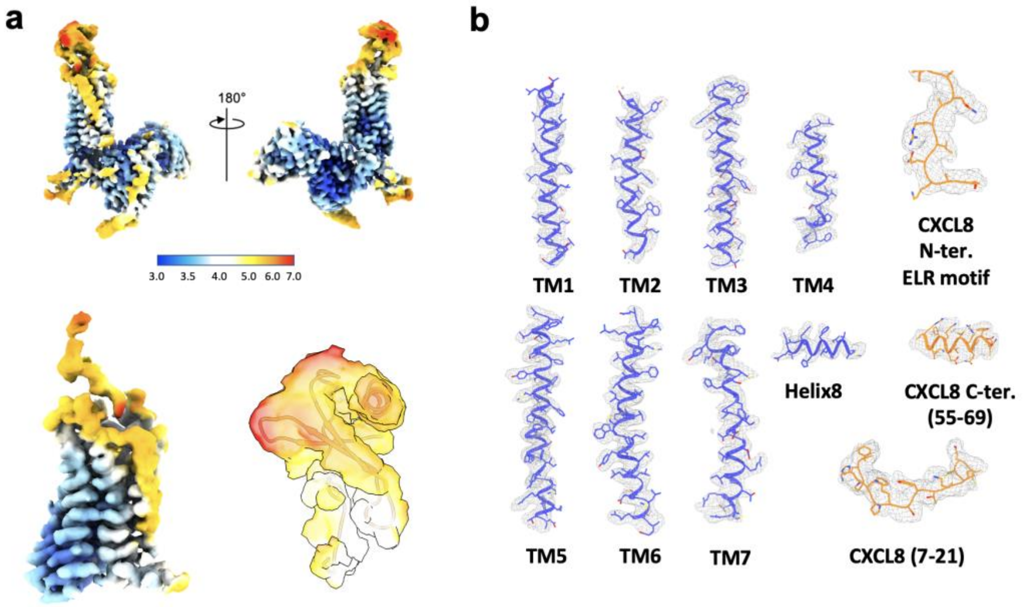

Supplementary Figure 3. **Local resolution and cryo-EM density maps of the refined structure of CXCR1/Gi/CXCL8$^{1\text{-}72}$ complex.** (a) Density map according to local resolution estimation. (b) The cryo-EM density maps covering transmembrane helices of the CXCR1 and CXCL8. The cryo-EM density maps are shown as countour levels of 0.04 and 0.01 for CXCR1 and CXCL8, repectively.

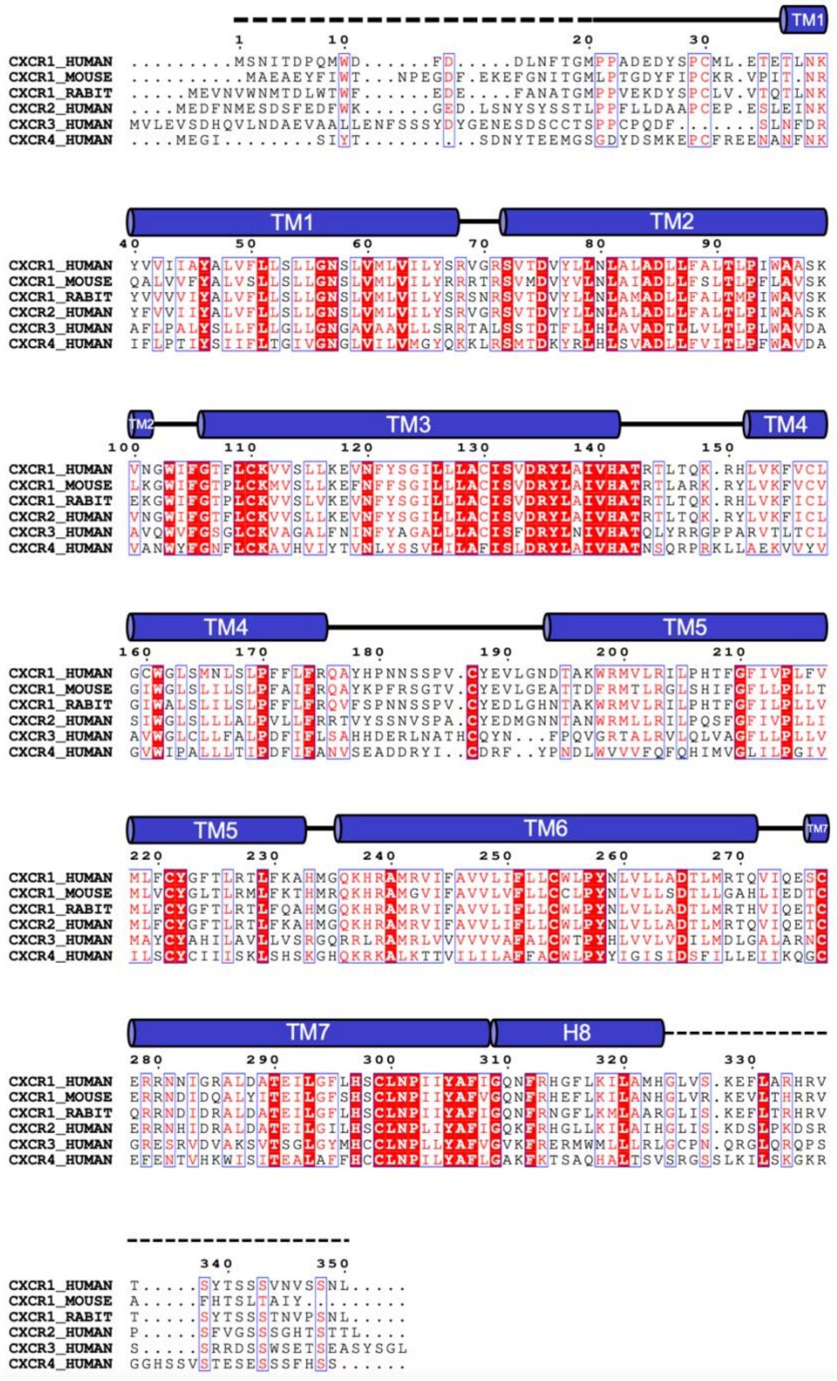

Supplementary Figure 4. **Sequence alignment of the CXC chemokine receptors**. Sequence alignment of CXC chemokine receptors calculated with CLUSTALW. The bars mark the locations of helices in CXCR1. Identical residues are shown in red, and similar residues are boxed.

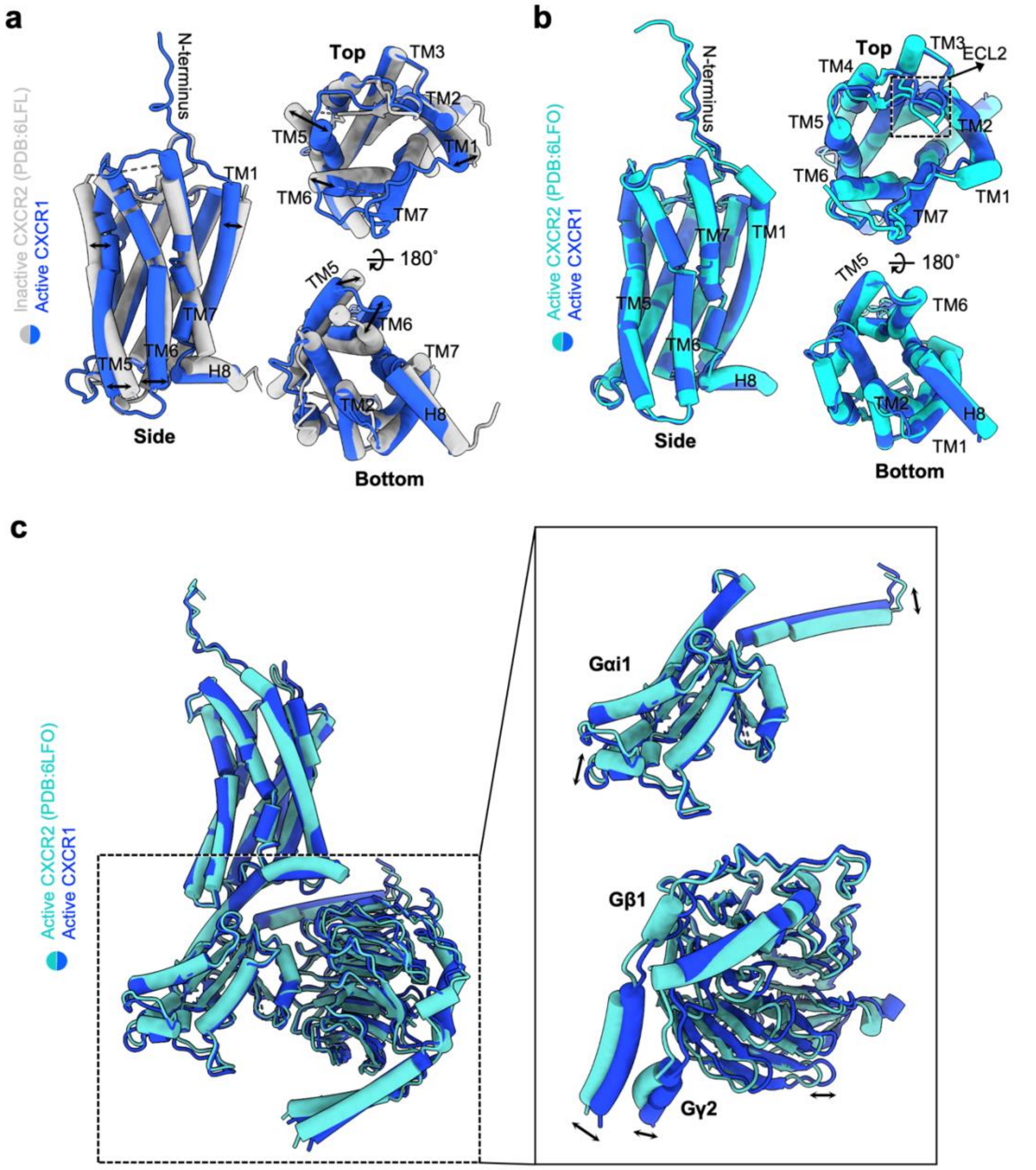

Supplementary Figure 5. **Structural comparison of CXCR1 and CXCR2.**
(a) Superposition of the active CXCR1 (blue) and inactive CXCR2 (grey). (b) Superposition of the active CXCR1 (blue) and active CXCR2 (cyan). Side, top and bottom views of the overlaid structures are displayed using cylinders to represent helices. The structural differences are indicated by arrows. (c) Superposition of the CXCR1/Gi (blue) and CXCR2/Gi complexes (cyan). The Gαi1 and Gβ1, Gγ2 subunits are enlarged in the right-hand panel. Black arrows indicate subtle movements of Gi heterotrimer.

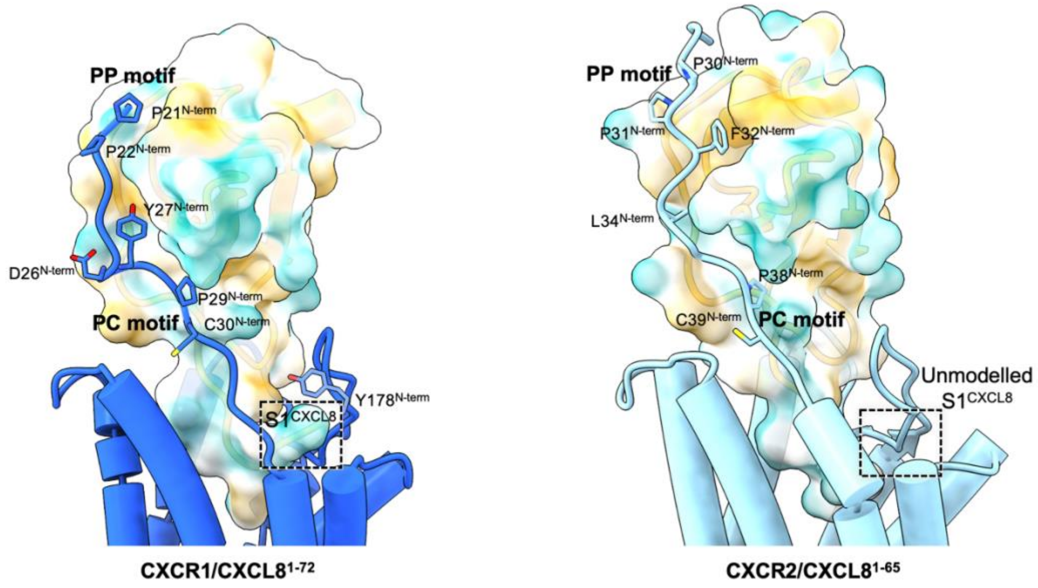

Supplementary Figure 6. **Comparison of CXCL8 interactions between CXCR1 and CXCR2.**

The interactions between receptor and CXCL8 in CRS1. Stick model and residue names are shown for conserved motifs (PP and PC motifs) and residues unique to CXCR1 and CXCR2 involved in CXCL8 binding. S1^{CXCL8} is indicated by a dashed box.

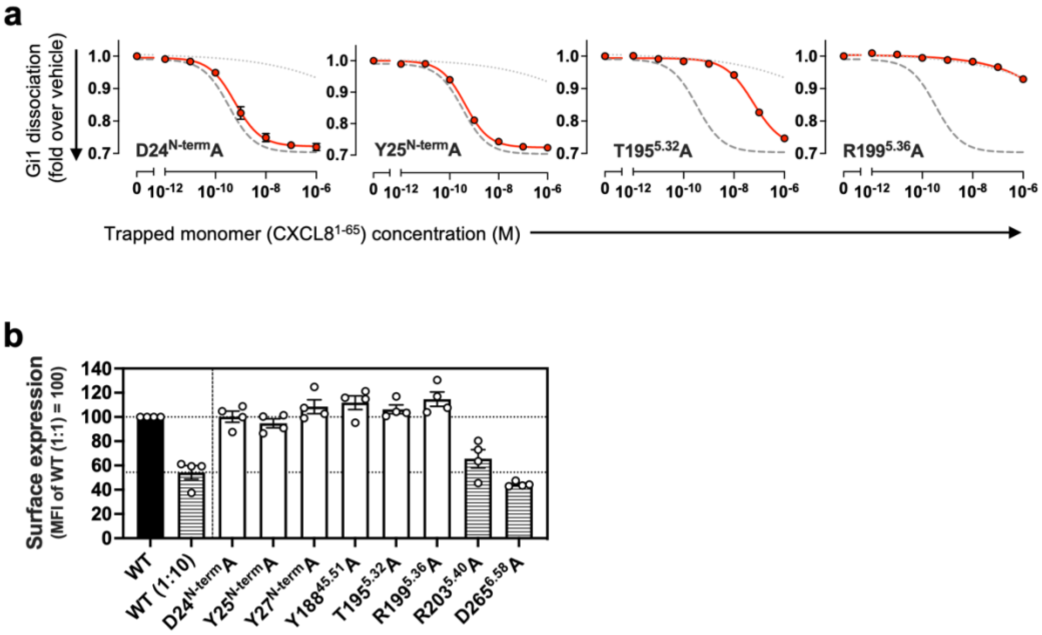

Supplementary Figure 7. **Functional assays and surface expression level of CXCR1 mutants.** (a) The NanoBiT-Gi-dissociation assay of the wild-type CXCR1 (gray dashed lines) and the mutants (red lines). Non-specific response in the mock-transfected cells is shown as gray dotted lines. Symbols and error bars represent mean and SEM, respectively, of three independent experiments. (b) Receptor expression levels on the cell surface were determined by flow cytometry with anti-FLAG antibody and their mean fluorescent intensity (MFI) was normalized to that of the wild-type performed in parallel. WT (1:10) represents a 10-fold less volume of transfected plasmid of WT (1:1). Bars and error bars represent mean and SEM, respectively, of four independent experiments.

Supplementary Table 1. Statistics for data collection and structural refinement

	CXCR1-Gi-scFv16-CXCL8
**PDB entry**	8IC0
**EMDB entry**	EMD-35351
**Data collection and processing**	
Magnification	105,000
Microscope	Titan Krios G4
Voltage (kV)	300
Detector	Gatan K3 Summit
Energy filter	Gatan Quantum-LS, 15 eV slit
Electric exposure (e^-/Å)	51.16
Deforcus range (μm)	-0.8 to -1.6
Collection mode	CDS mode
Pixel size (Å)	0.83
Data Processing Program	cryoSPARC (v.3.3.1)/Relion(v.4.0)
Movies	4,175
Initial / Final particle images (no.)	2,530,947 / 120,631
Symmetry imposed	C1
Map resolution (Å)	3.41
FSC threshold	0.143
**Refinement**	
Refinement Program	PHENIX (v.1.19.2)
Model resolution (Å)	3.36
FSC threshold	0.143
Model composition	
Non-hydrogen atoms	9,553
Protein residues	1,228
R.m.s. deviations	
Bond length (Å)	0.003
Bond angles (°)	0.550
Validation	
MolProbuty score	1.75
Clashscore	8.26
Ramachandran plot	
Favored / Allowed (%)	95.71 / 4.29
Disallowed (%)	0.00
Mask CC	0.75
