# Supplementary material for: Structural basis of CXC chemokine receptor 1 ligand binding and activation
Source: Nat Commun. 2023 Jul 11;14:4107. doi: 10.1038/s41467-023-39799-2 (PMC10336096; doi:10.1038/s41467-023-39799-2)
Supplement: Supplementary file 3 — Reporting Summary [file 41467_2023_39799_MOESM3_ESM.pdf]

nature portfolio

Corresponding author(s): Sam-Yong Park
Last updated by author(s): Jun 15, 2023

Reporting Summary

Nature Portfolio wishes to improve the reproducibility of the work that we publish. This form provides structure for consistency and transparency in reporting. For further information on Nature Portfolio policies, see our Editorial Policies and the Editorial Policy Checklist.

Statistics

For all statistical analyses, confirm that the following items are present in the figure legend, table legend, main text, or Methods section.

n/a	Confirmed	
☐	☒	The exact sample size (*n*) for each experimental group/condition, given as a discrete number and unit of measurement
☐	☒	A statement on whether measurements were taken from distinct samples or whether the same sample was measured repeatedly
☐	☒	The statistical test(s) used AND whether they are one- or two-sided<br>*Only common tests should be described solely by name; describe more complex techniques in the Methods section.*
☒	☐	A description of all covariates tested
☒	☐	A description of any assumptions or corrections, such as tests of normality and adjustment for multiple comparisons
☐	☒	A full description of the statistical parameters including central tendency (e.g. means) or other basic estimates (e.g. regression coefficient) AND variation (e.g. standard deviation) or associated estimates of uncertainty (e.g. confidence intervals)
☐	☒	For null hypothesis testing, the test statistic (e.g. *F*, *t*, *r*) with confidence intervals, effect sizes, degrees of freedom and *P* value noted<br>*Give P values as exact values whenever suitable.*
☒	☐	For Bayesian analysis, information on the choice of priors and Markov chain Monte Carlo settings
☒	☐	For hierarchical and complex designs, identification of the appropriate level for tests and full reporting of outcomes
☒	☐	Estimates of effect sizes (e.g. Cohen's *d*, Pearson's *r*), indicating how they were calculated

Our web collection on statistics for biologists contains articles on many of the points above.

Software and code

Policy information about availability of computer code

Data collection	EPU 2.9
Data analysis	cryoSPARC (v.3.3.1), Relion (v.4.0.0), COOT 0.9.8.7, PHENIX 1.19.2, GraphPad Prism 9, MassLynx 4.2, FlowJo software v10.9, ChimeraX 1.2.5, deepEMhancer v0.13

For manuscripts utilizing custom algorithms or software that are central to the research but not yet described in published literature, software must be made available to editors and reviewers. We strongly encourage code deposition in a community repository (e.g. GitHub). See the Nature Portfolio guidelines for submitting code & software for further information.

Data

Policy information about availability of data

All manuscripts must include a data availability statement. This statement should provide the following information, where applicable:

- Accession codes, unique identifiers, or web links for publicly available datasets
- A description of any restrictions on data availability
- For clinical datasets or third party data, please ensure that the statement adheres to our policy

The coordinates of the CXCR1/Gi/CXCL81-72 complex model have been deposited in the Protein Data Bank with the accession code 8IC0[https://doi.org/10.2210/pdb8ic0/pdb]. The cryo-EM map for CXCR1/Gi/CXCL81-72 complex has been deposited in the Electron Microscopy Data Bank with the accession code EMD-35351 [https://www.ebi.ac.uk/pdbe/entry/emdb/EMD-35351]. Previously published structures referenced can be found under accession codes 6LFL[https://doi.org/10.2210/pdb6lfl/pdb] (CXCR2/00767013 complex) and 6LFO[https://doi.org/10.2210/pdb6lfo/pdb] (CXCR2/Gi/CXCL81-68 complex), respectively. Source

data are provided with this paper.

Research involving human participants, their data, or biological material

Policy information about studies with human participants or human data. See also policy information about sex, gender (identity/presentation), and sexual orientation and race, ethnicity and racism.

Reporting on sex and gender	N/A
Reporting on race, ethnicity, or other socially relevant groupings	N/A
Population characteristics	N/A
Recruitment	N/A
Ethics oversight	N/A

Note that full information on the approval of the study protocol must also be provided in the manuscript.

Field-specific reporting

Please select the one below that is the best fit for your research. If you are not sure, read the appropriate sections before making your selection.

[x] Life sciences [ ] Behavioural & social sciences [ ] Ecological, evolutionary & environmental sciences

For a reference copy of the document with all sections, see nature.com/documents/nr-reporting-summary-flat.pdf

Life sciences study design

All studies must disclose on these points even when the disclosure is negative.

Sample size	Sample sizes were not predetermined for this study. The size of Cryo-EM data were determined by available time of microscope and the single particle density on grid.<br>For cell-based assay, at least three independent samples were used to ascertain the reproducibility and consistency of experiments.
Data exclusions	No data were excluded.
Replication	Cell-based experiments were repeated at least three times in independent experiments.<br>Experimental findings were reproduced reliably.<br>Structural analysis described in this study was not repeated. Replication is not necessary because structural analysis does not require verification of results and reproducibility by repeated experiments.
Randomization	Cryo-EM data are randomly divided as two sets (half maps) and used for resolution determination. Randomization is not required to other experiments because there are no unknown covariates.
Blinding	Investigators were not blinded. No blinding was needed for this study. Blinding is not necessary because experiments performed in this study do not require subject assessment of the data.

Reporting for specific materials, systems and methods

We require information from authors about some types of materials, experimental systems and methods used in many studies. Here, indicate whether each material, system or method listed is relevant to your study. If you are not sure if a list item applies to your research, read the appropriate section before selecting a response.

Materials & experimental systems

n/a	Involved in the study	
[ ]	[x]	Antibodies
[ ]	[x]	Eukaryotic cell lines
[x]	[ ]	Palaeontology and archaeology
[x]	[ ]	Animals and other organisms
[x]	[ ]	Clinical data
[x]	[ ]	Dual use research of concern
[x]	[ ]	Plants

Methods

n/a	Involved in the study	
[x]	[ ]	ChIP-seq
[ ]	[x]	Flow cytometry
[x]	[ ]	MRI-based neuroimaging

Antibodies

Antibodies used	In house antibody: scFv16 against Gαi protein<br>Commercial antibodies: Anti DYKDDDDK tag, Monoclonal Antibody (Clone 1E6, FujiFilm Wako Pure Chemicals, Catalog# 014-22383; 10 µg per ml diluted in 2% goat serum- and 2 mM EDTA-containing D-PBS (blocking buffer)), Goat anti-mouse IgG secondary antibody conjugated with Alexa Fluor 488 (Thermo Fisher Scientific, Catalog# A32723, ; 10 µg per ml diluted in the blocking buffer).
Validation	The commercial antibodies were validated by manufactures.<br>Anti DYKDDDDK tag, Monoclonal Antibody: Mouse/IgG2b (Host/Isotype) : Monoclonal anti DYKDDDDK tag antibody was established by fusing mouse myeloma cells and spleen cells of mice immunized with synthetic DYKDDDDK peptide which was coupled with keyhole limpet hemocyanin (KLH). This antibody was purified from mouse ascites.<br>Goat anti-mouse IgG secondary antibody conjugated with Alexa Fluor 488: Goat/IgG (Host/Isotype) : To minimize cross-reactivity, the goat anti-mouse IgG whole antibodies have been pre cross-adsorbed against bovine IgG, goat IgG, rabbit IgG, rat IgG, human IgG, and human serum. Cross-adsorption or pre-adsorption is a purification step to increase specificity of the antibody resulting in less background staining and cross-reactivity. The secondary antibody solution is passed through a column matrix containing immobilized serum proteins from potentially cross-reactive species. Only the nonspecific-binding secondary antibodies are captured in the column, and the highly specific secondaries flow through. Further passages through additional columns result in highly cross-adsorbed preparations of secondary antibody.

Eukaryotic cell lines

Policy information about cell lines and Sex and Gender in Research

Cell line source(s)	Sf9 (Thermo Fisher Scientific, A35243), HEK293A (Thermo Fisher Scientific, R70507).
Authentication	Sf9 and HEK293A cells were authenticated by growth properties and cell morphologies according to instruction provided by ThermoFisher.
Mycoplasma contamination	Not tested for mycoplasma contamination, but there are no indications of mycoplasma contamination.
Commonly misidentified lines<br>(See ICLAC register)	No commonly misidentified cell lines were used.

Flow Cytometry

Plots

Confirm that:

- [ ] The axis labels state the marker and fluorochrome used (e.g. CD4-FITC).
- [ ] The axis scales are clearly visible. Include numbers along axes only for bottom left plot of group (a 'group' is an analysis of identical markers).
- [ ] All plots are contour plots with outliers or pseudocolor plots.
- [ ] A numerical value for number of cells or percentage (with statistics) is provided.

Methodology

Sample preparation	HEK293A cells were seeded in a 6-cm culture dish at a concentration of 2 x 10 cells ml -1 (4 ml per well in DMEM (Nissui) supplemented with 10 % fetal bovine serum (Gibco), glutamine, penicillin and streptomycin), one day before transfection. Cells were grown overnight and transfected by transfection solution prepared by combining 10 µL (per dish hereafter) of polyethylenimine (PEI) Max solution (1 mg ml-1 ; Polysciences), 400 µL of Opti-MEM (Thermo Fisher Scientific) and a plasmid mixture containing desired plasmids. One day after transfection, the cells were collected by adding 200 µl of 0.53 mM EDTA-containing Dulbecco's PBS (D-PBS), followed by 200 µl of 5 mM HEPES (pH 7.4)-containing Hank's Balanced Salt Solution (HBSS). The cell suspension was transferred to a 96-well V-bottom plate in duplicate and fluorescently labeled with an anti-FLAG epitope (DYKDDDDK) tag monoclonal antibody (Clone 1E6, FujiFilm Wako Pure Chemicals; 10 µg per ml diluted in 2% goat serum- and 2 mM EDTA-containing D-PBS (blocking buffer)) and a goat anti-mouse IgG secondary antibody conjugated with Alexa Fluor 488 (Thermo Fisher Scientific, 10 µg per ml diluted in the blocking buffer). After washing with D-PBS, the cells were resuspended in 200 µl of 2 mM EDTA-containingD-PBS and filtered through a 40-µm filter.
Instrument	EC800 flow cytometer equipped with a 488 nm laser. The fluorescent signal derived from Alexa Fluor 488 was recorded in an FL1 channel.
Software	The flow cytometry data were analyzed with the FlowJo software
Cell population abundance	Values of mean fluorescence intensity (MFI) from approximately 20,000 cells per sample were used for analysis. For each experiment, we normalized an MFI value of the mutants by that of WT performed in parallel and denoted relative levels.
Gating strategy	Live cells were gated with a forward scatter (FS-Peak-Lin) cutoff at the 390 setting, with a gain value of 1.7.

- [ ] Tick this box to confirm that a figure exemplifying the gating strategy is provided in the Supplementary Information.
